# Epidemiological characteristics of occupational chemical poisonings in Zhejiang, China from 2006 to 2020: A descriptive analysis

**DOI:** 10.3389/fpubh.2022.999677

**Published:** 2022-11-16

**Authors:** Lifang Zhou, Fang Wei, Xinglin Fang, Yixin Zhang, Yong Hu, Xiaoming Lou, Panqi Xue, Hua Zou

**Affiliations:** ^1^Institute of Occupational Health and Radiation Protection, Zhejiang Provincial Center for Disease Control and Prevention, Hangzhou, China; ^2^Department of Public Health, Hangzhou Normal University, Hangzhou, China

**Keywords:** occupational disease, chemical poisoning, occupational hazards, epidemiological characteristics, descriptive analysis

## Abstract

**Objective:**

As the second most common occupational disease in China, occupational poisoning is one of the major public health problems that seriously affect workers' health. This study aimed to investigate the epidemiological and occupational characteristics of acute and chronic occupational poisoning cases in Zhejiang Province, so as to provide a scientific basis for proposing intervention measures and preventive strategies of occupational poisoning.

**Methods:**

The data on occupational poisoning cases in Zhejiang Province from 2006 to 2020 was derived from the National Occupational Disease Network Direct Report System. A descriptive statistical analysis was employed on this data utilizing R software.

**Results:**

From 2006 to 2020, 1,008 occupational poisoning cases were reported in Zhejiang Province, with a downward trend since 2007. Of these cases, 81.94% were chronic poisoning and 18.06% were acute poisoning. Ningbo reported the most occupational poisoning cases among the 11 cities in Zhejiang Province, accounting for 20.34% of the total cases. Besides, the occupational poisoning cases in Wenzhou, Jiaxing, and Shaoxing also accounted for 18.15%, 18.06%, and 17.76% of the total number of cases, respectively. Occupational poisoning in male were 693 cases and in female 315 cases. Most of the occupational poisoning cases studied involved people aged between 40 and 49 years (38.19%). The length of work in chronic occupational poisoning cases was significantly higher than that of acute occupational poisoning cases (*P* < 0.05). Benzene and lead and its compounds (excluding tetraethyl lead) were the major toxicants causing occupational poisoning. More than 60% of occupational poisoning cases were reported in private enterprises. Meanwhile, over 90% of the cases were distributed in medium enterprises and small enterprises. The type of industry with the most occupational poisoning cases was the manufacturing industry.

**Conclusion:**

Although the cases of occupational poisoning in Zhejiang Province have declined, more comprehensive and effective prevention and control measures are still needed. More attention ought to be paid to the management of key points according to the epidemiological and occupational characteristics of occupational poisoning cases.

## Introduction

Occupational poisoning, as the poisoning caused by exposure to toxic chemicals during work, is the occupational disease second only to pneumoconiosis in the Chinese occupational disease catalog (1, 2). In the National Occupational Disease Classification and Catalog revised in 2013 in China, a total of 60 toxicants (including one open item) causing occupational poisoning are listed (3). In addition to the inevitable use of toxic and dangerous chemicals in some workplaces, the high incidence of occupational poisoning is also influenced by multiple factors, such as outdated production processes, inadequate supervision, and management measures of enterprises, insufficient protective facilities in the workplace, inappropriate use of personal protective equipment, and workers' own weak safety awareness (4, 5).

Short-term high-concentration exposure of workers to toxicants in the workplace could lead to acute poisoning accidents, which can be fatal in severe cases (6, 7). Moreover, long-term exposure to low concentrations of toxicants is possible to cause chronic poisoning, inducing multi-organ dysfunction and even cancer (8–11). Among the occupational risks from the Global Burden of Disease Study 2017 (GBD 2017), occupational injuries, including occupational poisoning, were always the prime risk of attributable disability-adjusted life years (DALYs) in China from 1990 to 2017 (12). Occupational poisoning due to exposure to toxic chemicals has been a long-standing problem in the Chinese industry (5, 13). It is well-known that benzene and lead and its compounds endanger workers' health as the main chemicals that cause occupational poisoning in China (2). Benzene has an inhibitory effect on bone marrow after long-term exposure, and may increase the risk of developing non-Hodgkin's lymphoma (NHL), multiple myeloma (MM), and various other hematopoietic disorders (14). Moreover, lead damages many organs and systems in the body and can cause reproductive toxicity, neurotoxicity, carcinogenicity, hypertension, renal dysfunction, hematological effects, and so on (15). In recent years, occupational poisoning has become a public health issue that attracts more and more attention because it seriously endangers workers' health and social stability.

While occupational diseases have long been a concern, many countries lack official figures on occupational accidents and work-related illnesses due to the absence of proper recording and notification systems (16). In China, after the labor law and related policies were promulgated and implemented, in order to strengthen the surveillance for occupational diseases, the National Occupational Disease Network Direct Report System was established in 2006. Since then, under the requirements of the Ministry of Health, the diagnosis institutions have reported the information of confirmed occupational diseases diagnosis through the National Occupational Disease Network Direct Report System to manage the occupational disease report data through the network and computers (17).

Through the analysis of occupational poisoning cases in Zhejiang Province from 2006 to 2020 collected on the direct reporting system, this study elucidates the incidence trend and characteristics of occupational poisoning in Zhejiang Province in recent 15 years, so as to provide a scientific basis for the prevention and control strategies of occupational poisoning. Many enterprises in Zhejiang Province may use or produce various chemicals in the process of product manufacturing, which greatly increases the risk of occupational poisoning incidents. Therefore, clarifying the epidemiological characteristics of occupational poisoning in Zhejiang Province in the past 15 years is of great significance for decision-makers to effectively prevent occupational poisoning.

## Materials and methods

### Data source

The study utilized data on occupational poisoning cases from 1 January 2006 to 31 December 2020 in Zhejiang Province via the Occupational Disease and Health Hazards Monitoring System which is part of the China information system for disease control and prevention. All data has been confirmed, reviewed, and reported by occupational disease diagnosis institutions.

### Definition

The descriptive analysis of occupational poisoning cases reported in Zhejiang Province from 2006 to 2020 was carried out based on factors such as general information, region, types of toxicants, enterprise ownership type and scale, industry, and occupation.

Types of toxicants: 60 toxicants (including one open item) causing occupational poisoning are listed in the National Occupational Disease Classification and Catalog (3).

Enterprise ownership type: state-owned, collectively owned, privately owned, foreign, Hong Kong/Macao/Taiwan, and others (based on ownership of the major paid-in capital).

Enterprise scale: Based on the Enterprise Scale Standard established by the State Statistical Bureau (18), enterprises are divided into large, medium, small, and micro sizes.

Industry classification: The industry classification of the enterprises is based on the Industrial Classification for National Economic Activities (19), divided into 20 groups including 97 categories.

### Statistical analysis

The data on occupational poisoning in Zhejiang Province from 2006 to 2020 were introduced to Excel for sorting. Data management, analysis, and plotting were performed using R4.1.3. The continuous variables for the normal distribution were described as mean ± standard deviation (SD), and data with the skewed distribution were described using median and percentile (P_25_, P_75_). For the categorical variables, frequencies (*n*), and proportions (%) were calculated. After descriptive analysis of the data, further comparison between groups was performed using Chi-square test, Student's *t*-test and Wilcoxon rank sum test, and differences were considered statistically significant at *P* < 0.05. Mann-Kendall trend test was applied to determine whether the number of occupational poisoning cases over the years has a monotonic upward or downward trend, and *P* < 0.01 indicated a significant trend change.

## Results

From 2006 to 2020, a total of 1,008 occupational poisoning cases in Zhejiang Province were reported via the direct reporting system. Among these cases, chronic poisoning constituted 826 cases (81.94% of all cases), while cases involving acute poisoning were 182 (18.06%).

### Distribution of occupational poisoning by year

**Figure 1:**
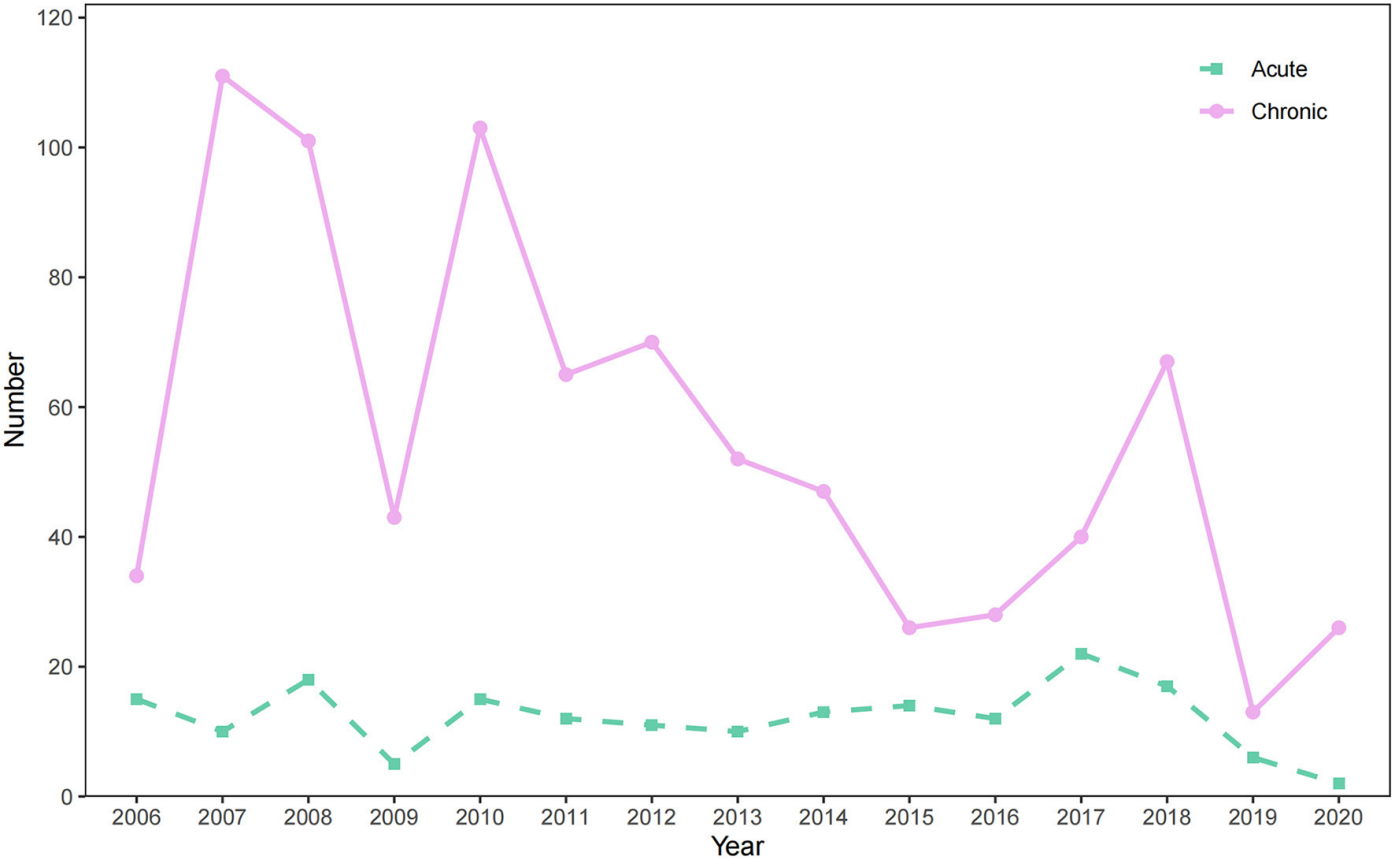
The changes in the number of occupational poisoning cases reported in Zhejiang Province from 2006 to 2020.

Figure 1 exhibits the evolution of occupational poisoning in Zhejiang Province, China between 2006 and 2020. The statistical differences in the number of poisoning cases between years were observed (χ^2^ = 49.24, *P* < 0.05). The number of occupational poisoning cases increased since 2006, and the growth rate from 2006 to 2007 reached 146.94%. From 2007 to 2010, the number of cases was generally at a high level, with a temporary decrease in 2009, after which the numbers rose again. From 2007 to 2020, although the number of occupational poisoning cases fluctuated, it showed a significant downward trend (*P* < 0.01). In particular, the trend of acute poisoning cases was relatively stable from 2006 to 2020, with a maximum of 22 cases in 2017. Chronic poisoning shows a similar trend to the overall occupational poisoning, with a maximum of 111 cases in 2007 and a significant downward trend since 2007 (*P* < 0.01).

### Regional distribution of occupational poisoning

**Figure 2:**
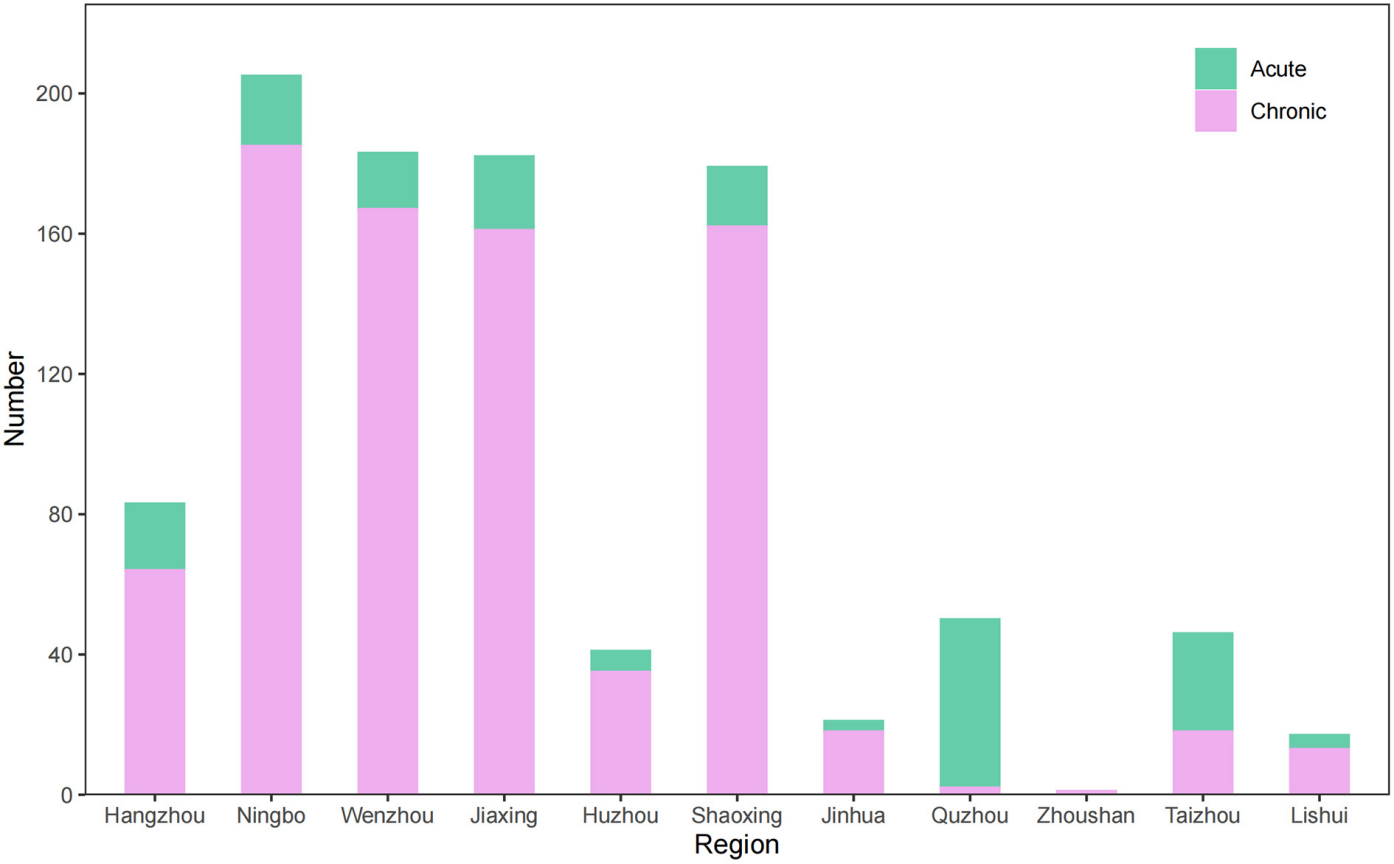
The regional distribution of occupational poisoning cases reported in Zhejiang Province.

From 2006 to 2020, occupational poisoning cases were reported in 11 cities in Zhejiang Province, of which Ningbo had the most with 205 cases (20.34%), followed by Wenzhou with 183 cases (18.15%), Jiaxing (182, 18.06%), Shaoxing (179, 17.76%), Zhoushan the least with only 1 case (0.10%) (Figure 2). The distribution of occupational poisoning cases in different regions showed statistically significant differences (χ^2^ = 299.06, *P* < 0.05). Occupational poisoning exhibited different characteristics in different regions. Acute poisoning was reported the most in Quzhou with 48 cases (26.37%), followed by Taizhou with 28 cases (15.38%), Jiaxing (21, 11.54%), and Ningbo (20, 10.99%). Chronic poisoning was reported the most in Ningbo with 185 cases (22.40%), followed by Wenzhou (167, 20.22%), Shaoxing (162, 19.61%), and Jiaxing (161, 19.49%).

According to the trend of occupational poisoning cases reported over the years, occupational poisoning in Shaoxing reached its peak in 2007 (74 cases), and then gradually declined and remained at a low level since 2010 (Supplementary Figure S1). The number of occupational poisoning cases in other cities fluctuated with no obvious trend over the years.

### Population characteristics of occupational poisoning

**Figure 3:**
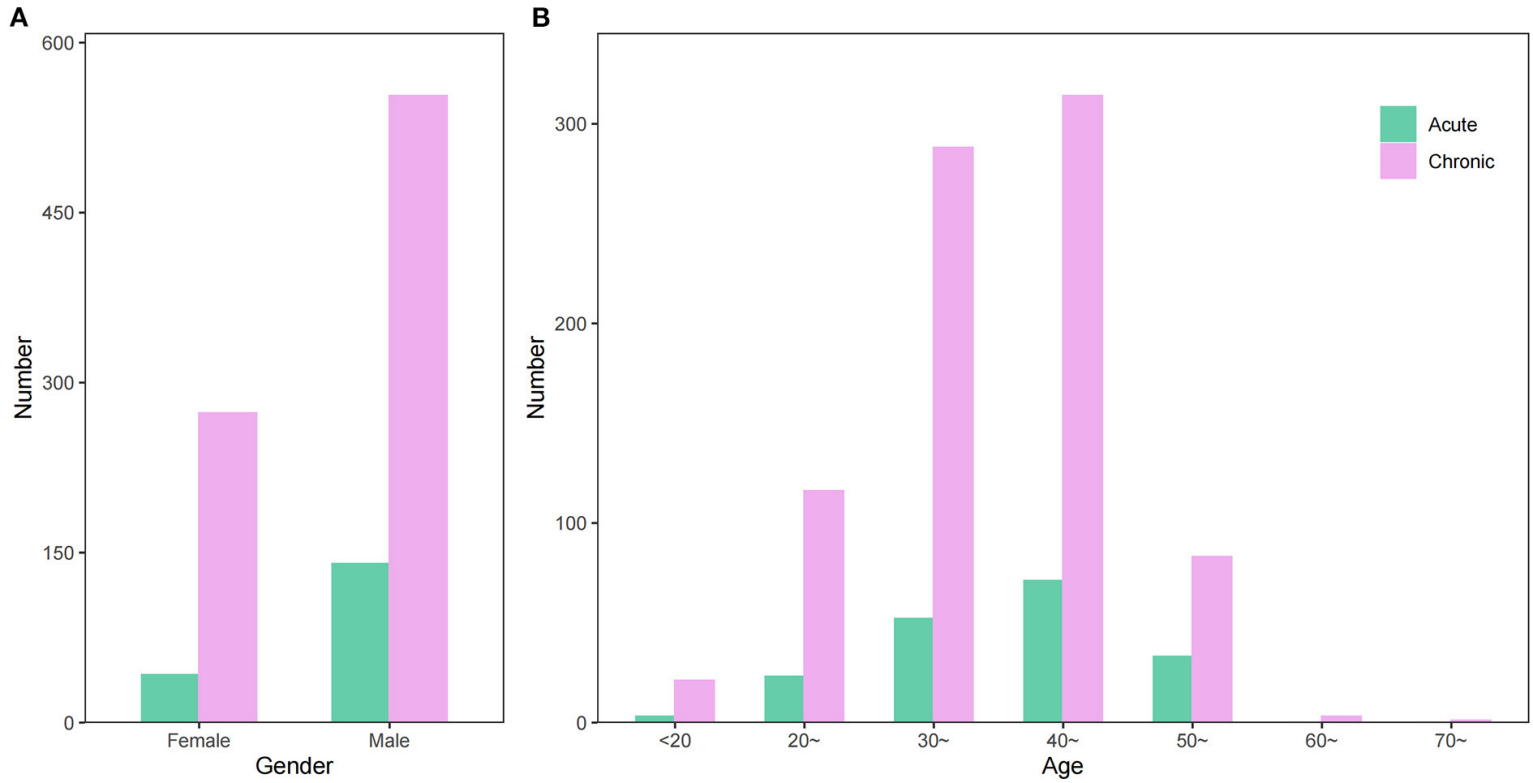
The gender **(A)** and age distribution **(B)** of occupational poisoning cases reported in Zhejiang Province from 2006 to 2020.

From 2006 to 2020, 693 (68.75%) occupational poisoning cases in males and 315 (31.25%) in females were reported in Zhejiang Province, among which the number of acute poisoning cases and chronic poisoning cases in males was 3.33 times and 2.03 times that of females, respectively. The number of male and female poisoning cases differed in a statistically significant manner (χ^2^ = 6.45, *P* < 0.05). The average age of poisoning cases was 38.83 ± 9.28, of which the age of acute poisoning cases and chronic poisoning cases were 40.51 ± 9.75 and 38.46 ± 9.15, respectively. A statistical difference was found between the age of acute and chronic poisoning cases (*t* = 2.59, *P* < 0.05). The acute and chronic occupational poisoning cases were both primarily found in the age group of 40–49 years, accounting for 39.01% and 38.01% of all acute and chronic poisoning cases, respectively (Figure 3).

**Figure 4:**
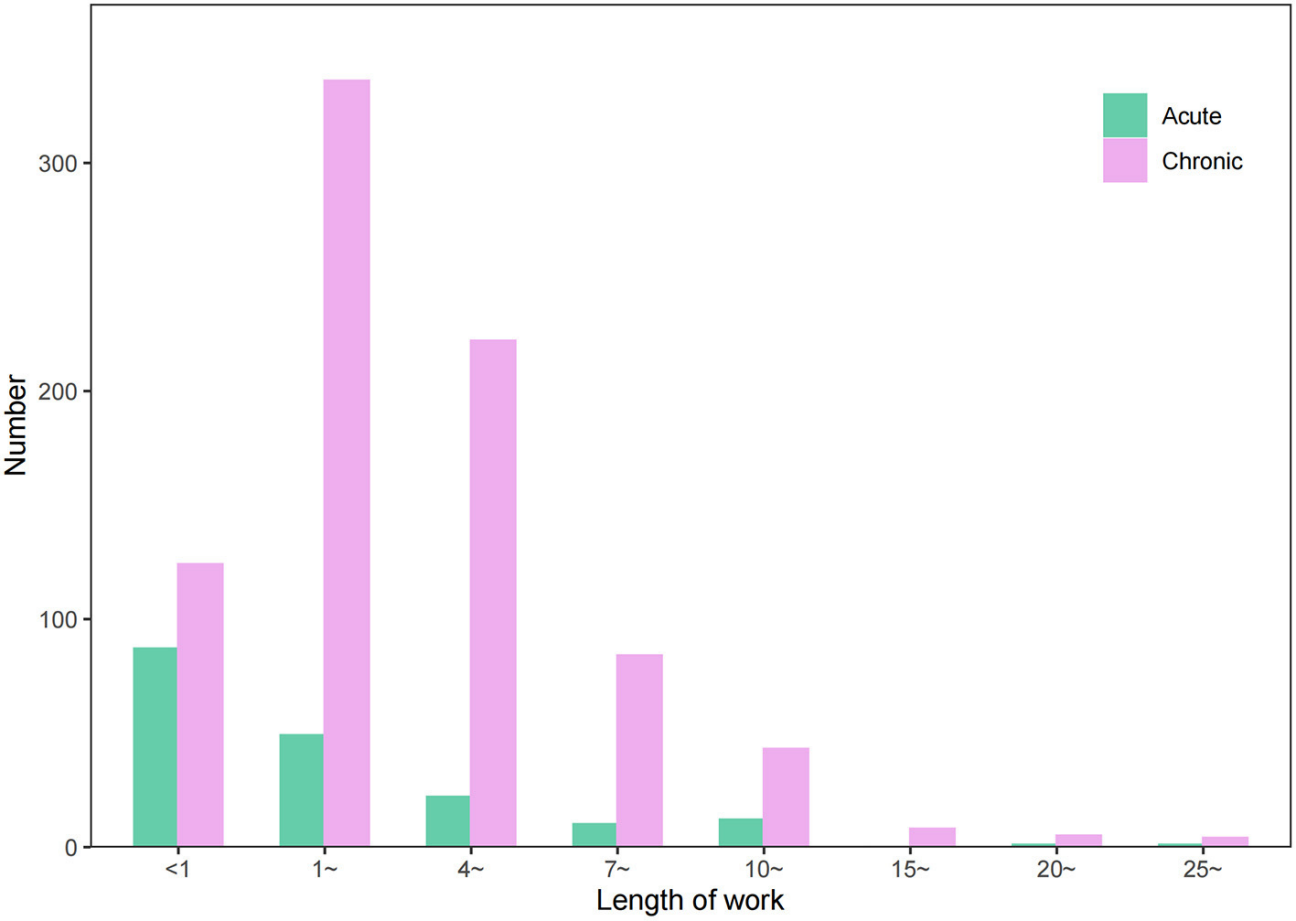
The length of work of occupational poisoning cases reported in Zhejiang Province from 2006 to 2020.

From 2006 to 2020, the median length of work [*M* (P_25_, P_75_)] at the onset time of occupational poisoning cases in Zhejiang Province was 3.25 (1.25, 6.00) years. The length of work in chronic occupational poisoning cases was 3.50 (1.67, 6.17) years, which was significantly higher than that of acute occupational poisoning cases with 1.06 (0.27, 3.94) years (*P* < 0.05). The acute occupational poisoning cases were mainly distributed in the group of work length at 0–1 years with 87 cases (47.80%), while the group of work length with the most chronic occupational poisoning cases was 1–4 years (336 cases), accounting for 40.68% of the total number of chronic poisoning cases (Figure 4).

### Types of toxicants causing occupational poisoning

**Figure 5:**
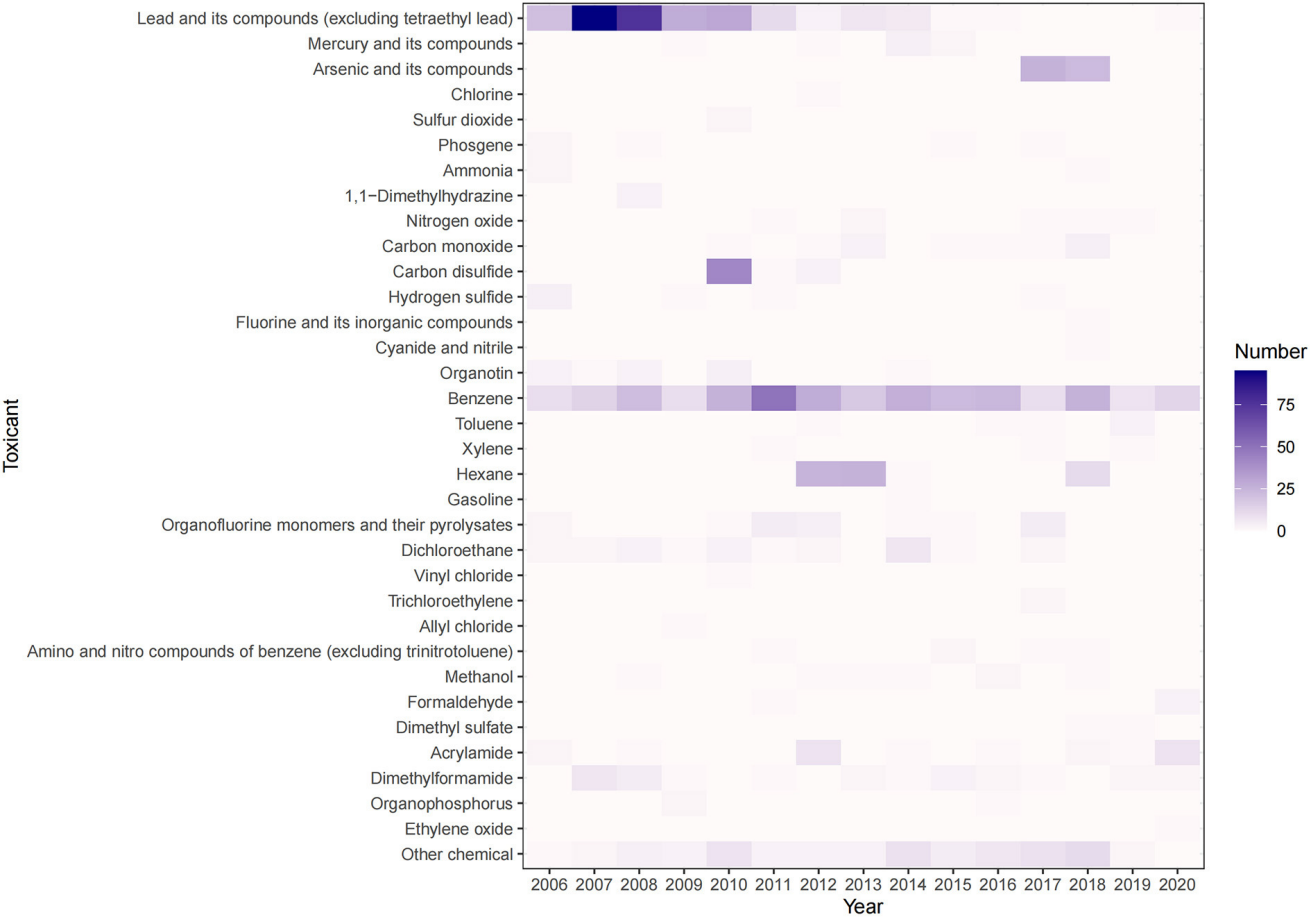
Types of toxicants causing occupational poisoning in Zhejiang Province from 2006 to 2020.

From 2006 to 2020, there were differences between the number of cases of different toxicants (*P* < 0.05), where the major toxicant that caused occupational poisoning were benzene with 309 cases (30.65%), and lead and its compounds (excluding tetraethyl lead) with 280 cases (27.78%). As shown in Figure 5, Benzene poisoning cases increased in 2011 (50 cases) and fluctuated around 20 in the remaining years. Lead and its compounds (excluding tetraethyl lead) poisoning cases increased in 2007 (95 cases) and 2008 (76 cases), and then gradually decreased to <1 case per year since 2015. Poisoning cases of arsenic and its compounds increased in 2017 (26 cases) and 2018 (23 cases), and no cases were reported in the remaining years. Carbon disulfide poisoning cases increased in 2010 (42 cases), and almost no cases were found in the remaining years. Hexane poisoning cases increased in 2012 (25 cases), 2013 (26 cases), and 2018 (11 cases), with almost no cases in the remaining years. The trends of some poisoning cases fluctuated in waves, such as acrylamide, dichloroethane, dimethylformamide, organofluorine monomers, and their pyrolysates, etc., while the cases of other toxicants had relatively stable trends (Supplementary Figure S2).

### Enterprise ownership type, enterprise scale, and industry distribution of occupational poisoning cases

Between 2006 and 2020, there were differences between the number of poisoning cases in different ownership types of enterprises (χ^2^ = 93.43, *P* < 0.05), with most of the occupational poisoning cases occurring in private enterprises and foreign enterprises, whose were 683 (67.76%) and 196 (19.44%), respectively. The enterprises with other ownership types reported 129 cases (12.80%) (Table 1). There were 74.73% of acute poisoning cases and 66.22% of chronic poisoning cases reported in private enterprises.

**Table 1:** The enterprise ownership type distribution of occupational poisoning cases reported from 2006 to 2020.

**Enterprise ownership**	**Acute poisoning**		**Chronic poisoning**		**Total**	
	***n***	**%**	***n***	**%**	***n***	**%**
State-owned	24	13.19	9	1.09	33	3.27
Collective	8	4.40	42	5.08	50	4.96
Private	136	74.73	547	66.22	683	67.76
Foreign	10	5.49	186	22.52	196	19.44
Hong Kong, Macao, and Taiwan	2	1.10	25	3.03	27	2.68
Other types	2	1.10	17	2.06	19	1.88

Statistically significant differences were also found in the occupational poisoning cases occurring in enterprises of different sizes (χ^2^ = 70.85, *P* < 0.05). Among the occupational poisoning cases, there were 42 cases (4.17%) in large enterprises, 551 cases (54.66%) in medium enterprises, 399 cases (39.58%) in small enterprises, 2 cases (0.20%) in micro enterprises, and 14 cases (1.39%) in enterprises with unknown size. Among them, the small enterprises had the most acute poisoning cases (121 cases, 66.48%); the medium enterprises had the most chronic poisoning cases (497 cases, 60.17%) (Table 2).

**Table 2:** The enterprise scale distribution of occupational poisoning cases reported from 2006 to 2020.

**Enterprise Size**	**Acute poisoning**		**Acute poisoning**		**Total**	
	***n***	**%**	***n***	**%**	***n***	**%**
Large	6	3.30	36	4.36	42	4.17
Medium	54	29.67	497	60.17	551	54.66
Small	121	66.48	278	33.66	399	39.58
Micro	1	0.55	1	0.12	2	0.20
Unknown	0	0.00	14	1.69	14	1.39

**Figure 6:**
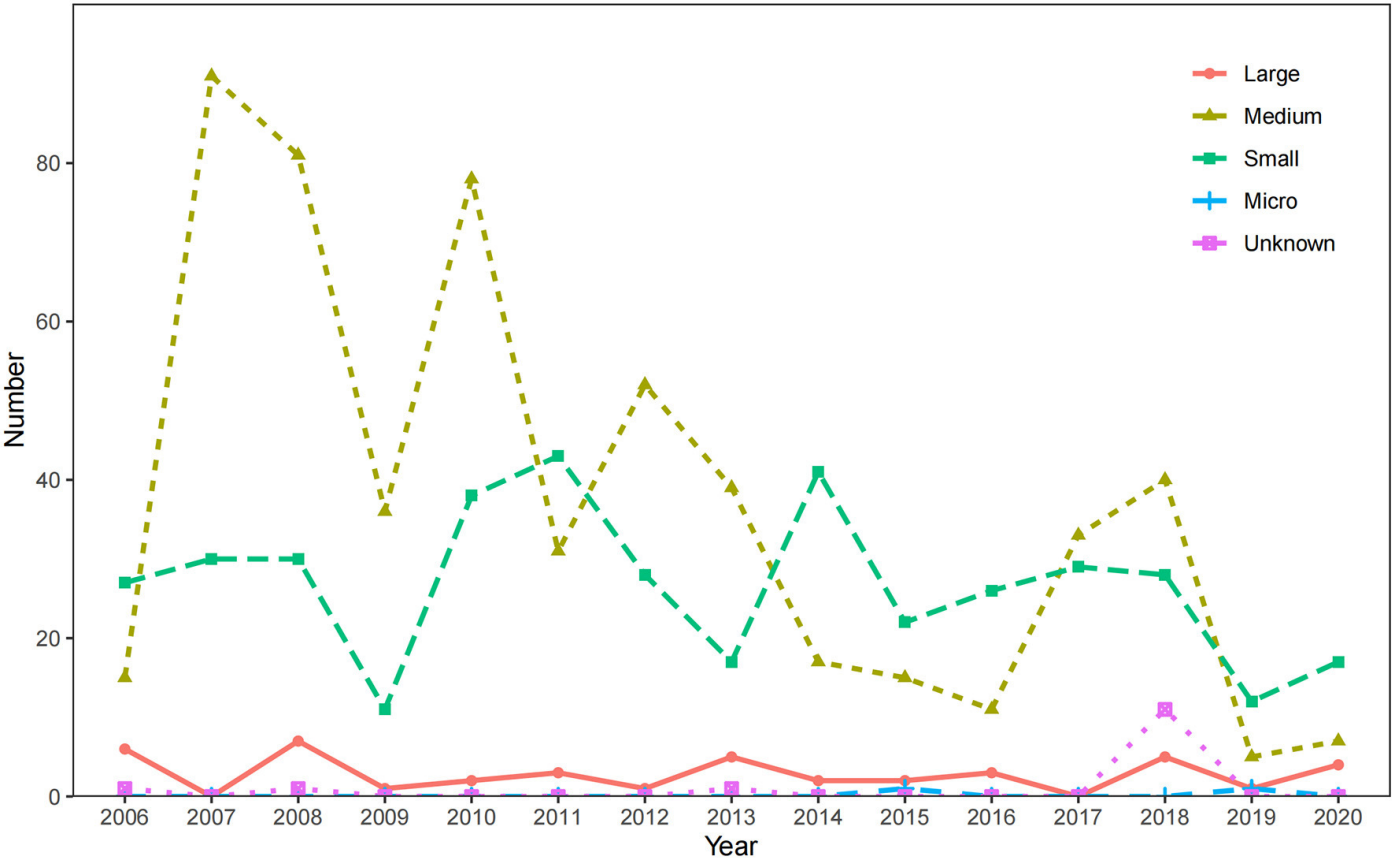
The changes in the number of occupational poisoning cases in enterprises of different scales from 2006 to 2020.

The curve of poisoning cases from medium enterprises and small enterprises fluctuated between 2006 and 2020 (Figure 6). Medium enterprises had the most occupational poisoning cases in 2007 (91 cases) and the least in 2019 (5 cases); small enterprises had the most in 2011 (43 cases) and the least in 2009 (11 cases). The number of cases in enterprises of other sizes was relatively stable over the years.

In terms of industry distribution, all the occupational poisoning cases reported in Zhejiang Province from 2006 to 2020 were distributed in 13 industries. The number of cases varied by industry (χ^2^ = 41.54, *P* < 0.05), with cases mainly distributed in the manufacturing industry, accounting for more than 90% (Supplementary Table S1).

## Discussion

Although the number of reported occupational poisoning cases is limited, as one of the most common occupational diseases in Zhejiang Province only second to pneumoconiosis (20), the incidence of occupational poisoning is affected by multiple factors and often leads to adverse consequences. Based on the data of occupational poisoning cases reported in Zhejiang Province from 2006 to 2020, we analyzed the epidemiological and occupational characteristics of occupational poisoning in the past 15 years. From 2006 to 2020, the occupational poisoning cases reported in Zhejiang Province were dominated by chronic poisoning, which was consistent with studies in Jiangsu Province, Guangdong Province, and China (5, 13, 21, 22). The incidence trend increased greatly from 2006 to 2007, and then a downward trend was observed from 2007 to 2020. The increase in reported cases from 2006 to 2007 was related to the fact that many occupational poisoning patients were intensively reported by medical institutions after China launched the network direct report system of occupational diseases in 2006 (17). The number of cases gradually declined in trend since 2007, but attention still should be paid to the slight rebound in recent years. From 2006 to 2015, the proportion of acute poisoning cases accounted for 15.87% of the total occupational poisoning cases in Zhejiang Province, which was between 9.20% in Tianjin and 35.82% in Jiangsu Province during the same period (22, 23).

The occupational poisoning cases in this study were dominated by males, similar to the proportion of male and female workers in Zhejiang Province (24). Although the proportion of occupational poisoning in males is higher than that in females, the proportion in females still exceeds 30%, suggesting that we should also pay attention to the health of female workers. More than 70% of the poisoning cases are young and middle-aged (30–50 years old), and more than 80% are those with the length of work <7 years, suggesting that we should carry out health education and reasonable interventions for these groups. The chronic poisoning cases usually occur in workers with 1–7 years of work length, and health monitoring should be taken for them. The acute poisoning cases have shorter work length, mainly <1 year of work length, which may be related to improper operation, inadequate personal protection, and lack of experience in emergency treatment.

More than 74% of occupational poisoning cases were distributed in Ningbo, Wenzhou, Jiaxing, and Shaoxing. Most cities had more chronic poisoning cases than acute poisoning cases, with the exception of Quzhou and Taizhou, where the number of acute poisoning cases was greater than that of chronic poisoning cases. Acute poisoning often occurs in the chemical industry, and Quzhou, where the incidence of acute occupational poisoning in this study is high, has developed a fluorine silicon industrial cluster over the years, and the chemical industry is a pillar industry in Quzhou (25, 26). In Zhejiang Province, Quzhou is relatively less economically developed thus the number of occupational poisoning cases is less, but the cases are mostly acute poisoning probably due to the backward technology of production, low investment in occupational health prevention and control of enterprises, and the lack of management measures. In comparatively, Zhoushan has many islands, and the chemical industry is less developed, so occupational poisoning occurs less often. The regional distribution of occupational poisoning cases was closely related to the population size, the industry structure, and the ability to prevent and control the incidence of occupational poisoning in a region. Therefore, these cities should be regarded as the key regions for occupational poisoning prevention and control in Zhejiang Province.

There are many types of toxicants that cause occupational poisoning, and the types of toxicants that cause acute and chronic poisoning are diverse. In this study, benzene and lead and its compounds (excluding tetraethyl lead) were the major toxicants of chronic poisoning, and they were also the toxicants that caused the most occupational poisoning cases, accounting for more than 50% of the total occupational poisoning cases. A study in 2018 by Wang et al. (2) demonstrated that lead and its compounds (except tetraethyl lead) and benzene were the leading causes of occupational poisoning in China, accounting for 31.30% and 26.76% of the total reported cases from 2010 to 2014, respectively. These results are close to the data of this study. Benzene poisoning cases were mainly distributed in Wenzhou, Ningbo, and Jiaxing, with 100, 59, and 56 cases, respectively (Supplementary Figure S3). According to the trend of benzene poisoning cases over the years, the number of benzene poisoning cases reported in 2011 was the largest, among which Wenzhou reported 36 cases, accounting for 72.0% of the benzene poisoning cases in that year (Supplementary Figure S4). Subsequently, there were sporadic cases in various cities from 2012 to 2020, with 8–28 cases per year. Benzene or benzene-containing solvents and adhesives are widely used in the shoemaking and leather products and luggage manufacturing industries in China. Accordingly, in this study, benzene poisoning also occurred mainly in industries such as leather products and shoemaking, consistent with the study by Liang et al. (27). The poisoning cases of lead and its compounds (excluding tetraethyl lead) were mainly distributed in Shaoxing and Ningbo, with 149 and 83 cases, respectively (Supplementary Figure S3). Based on the lead poisoning cases reported in 11 cities in Zhejiang Province from 2006 to 2020, Shaoxing reported the most lead poisoning cases in 2007, reaching 74 cases, and then decreased year by year (Supplementary Figure S4). The growth of lead-acid battery manufacturing industry has brought lead pollution (28), and the occurrence of chronic lead poisoning in this study is primarily attributable to the rapid development of the lead-acid battery manufacturing industry in Zhejiang Province in recent years. In addition, hexane, arsenic and its compounds, carbon disulfide were also toxicants that frequently caused chronic poisoning cases and were clustered in some regions and industries. Forty-nine poisoning cases of arsenic and its compounds were reported in Wenzhou, 46 poisoning cases of hexane and 46 poisoning cases of carbon disulfide were reported in Jiaxing, respectively. The poisoning cases of these chemicals in these cities were far higher than in others (Supplementary Figure S3). Organic compounds such as dimethylformamide, dichloroethane, organofluorine monomers, and their pyrolysates were the main toxicants causing acute poisoning, with sporadic occurrences between 2006 and 2020 (Supplementary Figure S2).

In recent years, the occurrence of lead and benzene occupational poisoning was severe in Zhejiang Province. Previous studies have shown that most organic solvents have chronic toxic effects, among which benzene is a known carcinogen in humans (29, 30). Regarding lead poisoning, it has been demonstrated that lead, as a cumulative toxicant, has detrimental effects on different organ systems, even in low doses (31, 32). In particular, benzene is a common and major toxicant in occupational poisoning in Zhejiang Province, and benzene poisoning in chronic poisoning still needs to be paid attention to. In addition, the number of lead poisoning cases was second only to benzene poisoning, but lead poisoning has declined to no more than one case per year since 2015, indicating that occupational poisoning caused by lead has been effectively controlled.

There are many types of enterprises in Zhejiang Province, and many types and quantities of chemicals are used in production (33). This study also demonstrated that the reported poisoning cases were mainly composed of poisoning cases in medium and small private enterprises, among which chronic poisoning was dominated by medium enterprises, while the main type of enterprise scale of acute poisoning cases was small. The results were similar to that of previous studies on occupational poisoning in Jiangsu (34). There are a multitude of medium and small enterprises in Zhejiang Province, and these enterprises have contributed significantly to the social and economic development of Zhejiang Province. However, most of them are still restricted by traditional industries, or are emerging industries but the manufacturing process of enterprises is backward (35). At the same time, the absence of effective protective facilities and measures in the workplace and the lack of standardized management of occupational disease prevention and control of enterprises enables occupational poisoning to be prevalent in the workplace, and the development of enterprises is under increasing pressure (22).

A wide range of toxic chemicals is in heavy use, and numerous new technologies, new materials, and new products are continuously introduced in Zhejiang Province in recent years (36). Besides, a considerable number of private and small enterprises were distributed in Zhejiang Province (35), but the expenditure on occupational disease prevention and control by private and small enterprises was insufficient (37). More importantly, effective engineering controls were not applied in the workplace. Meanwhile, no or inadequate personal protective equipment was in use, and inadequate hazard communication training was organized for exposed workers (38). Moreover, due to the low frequency of occupational health checks in workers and the limited range of diseases in the occupational diseases list, the actual burden of occupational diseases including occupational poisoning in China is likely to be underestimated (39). Under the circumstances, the workers were at increasing risk of occupational chemical poisoning (20). Where practicable, engineering and work practice controls ought to be utilized as the predominant means to reduce workers' exposure to toxic chemicals. As the crucial control techniques, engineering control techniques should be used in the workplace to minimize workers' exposure to hazardous substances like benzene and lead and its compounds in this study, such as optimizing processes to decrease exposure to harmful chemicals, ventilating to dilute the concentration of hazardous substances in the working environment, and decontaminating and recycling poisons (40, 41).

Industry distribution showed that occupational poisoning cases in Zhejiang Province were mainly concentrated in the manufacturing industry. Zhejiang Province is one of the earliest developing provinces of manufacturing industry in China. Since the late 1970s, after decades of rapid development, the manufacturing industry in Zhejiang Province has now developed into an important domestic manufacturing agglomeration and export base for industrial manufactured products (42). The sum of chronic poisoning cases in the three manufacturing industries accounted for nearly 50% of the total number of chronic poisoning cases, including electric machinery and equipment manufacturing industry; manufacturing industry of computers, communication, and other electronic equipment; manufacturing industry of leather, fur, feather and their products, and shoes. In these three industries, lead and organic solvents such as benzene are commonly used in the production process (27, 28, 43, 44). Insufficient use of protective equipment for workers can easily lead to poisoning. Acute poisoning mainly occurred in the chemical feedstock and chemical manufacturing industry, where the incidence of chronic poisoning was also relatively high (Supplementary Table S1). Therefore, for these key industries where occupational poisoning may occur, various measures such as improving rules and regulations should be taken to reduce the exposure level of workers to chemical hazards and protect workers' health.

## Conclusion

Occupational chemical poisoning is a category of occupational diseases with high fatality rate and great harm to workers. In this study, epidemiological and occupational characteristics of occupational poisoning cases in Zhejiang Province from 2006 to 2020 were described. The results exhibited that occupational poisoning cases reported in Zhejiang Province were predominantly chronic poisoning, and acute poisoning occasionally occurred. According to the characteristics of occupational poisoning cases, classified prevention and control strategies should be implemented, and the key points should be highlighted. Lead and benzene poisoning should be listed as key occupational diseases for surveillance, and the control of these toxicants should be strengthened. Great importance should be attached to private small and medium-sized enterprises in regions with a high incidence of occupational poisoning, and the prevention and treatment of occupational poisoning shall be carried out according to the characteristics of industry distribution.

## Data availability statement

The original contributions presented in the study are included in the article/Supplementary material, further inquiries can be directed to the corresponding authors.

## Ethics statement

The studies involving human participants were reviewed and approved by Ethics Committee of Zhejiang Provincial Center for Disease Control and Prevention (2022-026-01). The patients/participants provided their written informed consent to participate in this study.

## Author contributions

LZ: conceptualization, investigation, formal analysis, and writing original draft. FW: data curation, methodology, and formal analysis. XF: data curation and funding acquisition. YZ: formal analysis. YH: data curation and supervision. XL: conceptualization and supervision. PX: conceptualization, data curation, visualization, and writing—review and editing. HZ: methodology, writing—review and editing, funding acquisition, and supervision. All authors contributed to the article and approved the submitted version.

## Funding

This work was supported by the Medical Health Technology Project by the Health Commission of Zhejiang (Grant Number: 2019KY056), 2021 Zhejiang Provincial Center for Disease Control and Prevention for the Technology Talent Incubation Project.

## Conflict of interest

The authors declare that the research was conducted in the absence of any commercial or financial relationships that could be construed as a potential conflict of interest.

## Publisher's note

All claims expressed in this article are solely those of the authors and do not necessarily represent those of their affiliated organizations, or those of the publisher, the editors and the reviewers. Any product that may be evaluated in this article, or claim that may be made by its manufacturer, is not guaranteed or endorsed by the publisher.
