# Supplementary material for: Epidemiological characteristics of occupational chemical poisonings in Zhejiang, China from 2006 to 2020: A descriptive analysis
Source: Front Public Health. 2022 Nov 16;10:999677. doi: 10.3389/fpubh.2022.999677 (PMC9709323; doi:10.3389/fpubh.2022.999677)
Supplement: Supplementary file 1 [file Data_Sheet_1.PDF]

Supplementary Material

Table S1. The industry distribution of occupational poisoning cases reported in Zhejiang Province from 2006 to 2020.

Industry	Acute		Chronic		Sum	
	n	%	n	%	n	%
Farming, forestry, animal husbandry and fishery	1	0.55	0	0.00	1	0.10
Mining industry	0	0.00	51	6.17	51	5.06
Manufacturing industry	165	90.66	747	90.44	912	90.48
Manufacturing of agricultural and non-staple foodstuff	0	0.00	1	0.12	1	0.10
Textile industry	2	1.10	10	1.21	12	1.19
Textile costumes manufacturing industry	0	0.00	1	0.12	1	0.10
Manufacturing industry of leather, fur, feather and their products and shoes	21	11.54	101	12.23	122	12.10
Wood processing and manufacturing industry of wood, bamboo, rattan, palm, and straw-made articles	0	0.00	4	0.48	4	0.40
Cabinetmaking industry	1	0.55	51	6.17	52	5.16
Papermaking and paper product industry	5	2.75	1	0.12	6	0.60
Printing industry and reproduction of record media	1	0.55	19	2.30	20	1.98
Manufacturing industry for culture, education, artwork, sports and recreational goods	0	0.00	8	0.97	8	0.79
Petroleum, coal and other fuel processing industry	1	0.55	4	0.48	5	0.50
Chemical feedstock and chemical manufacturing industry	65	35.71	68	8.23	133	13.19
Medicine manufacturing industry	10	5.49	6	0.73	16	1.59
Chemical fiber manufacturing industry	0	0.00	49	5.93	49	4.86
Rubber and plastic production industry	15	8.24	18	2.18	33	3.27
Non-metallic minerals product industry	5	2.75	6	0.73	11	1.09
Ferrous metal smelting and extrusion industry	1	0.55	4	0.48	5	0.50
Non-ferrous smelting and extrusion industry	3	1.65	4	0.48	7	0.69
Metalwork industry	10	5.49	25	3.03	35	3.47
General-purpose equipment manufacturing industry	3	1.65	25	3.03	28	2.78
Specialized facility manufacturing industry	13	7.14	13	1.57	26	2.58
Automotive manufacturing industry	3	1.65	9	1.09	12	1.19

Industry	Acute		Chronic		Sum	
	n	%	n	%	n	%
Railway, marine, aerospace and other transportation equipment manufacturing industry	0	0.00	7	0.85	7	0.69
Electric machinery and equipment manufacturing industry	1	0.55	182	22.03	183	18.15
Manufacturing industry of computers, communication and other electronic equipment	1	0.55	125	15.13	126	12.50
Manufacturing industry of instruments and meters	1	0.55	0	0.00	1	0.10
Other manufacturing industries	1	0.55	5	0.61	6	0.60
Comprehensive utilization industry of waste resources	2	1.10	1	0.12	3	0.30
Production and supply of electric power, gas and water	2	1.10	0	0.00	2	0.20
Construction industry	2	1.10	2	0.24	4	0.40
Wholesale and retail trade	1	0.55	7	0.85	8	0.79
Traffic, storage and mail business	3	1.65	4	0.48	7	0.69
Accommodation and food industry	0	0.00	1	0.12	1	0.10
Leasehold and business service industry	4	2.20	5	0.61	9	0.89
Scientific research and technical service industry	2	1.10	3	0.36	5	0.50
Water conservancy, environment and public institution management	1	0.55	0	0.00	1	0.10
Neighborhood services and other service industry	1	0.55	5	0.61	6	0.60
Sanitation, social security and social welfare industry	0	0.00	1	0.12	1	0.10
Sum	182	100.00	826	100.00	1008	100.00

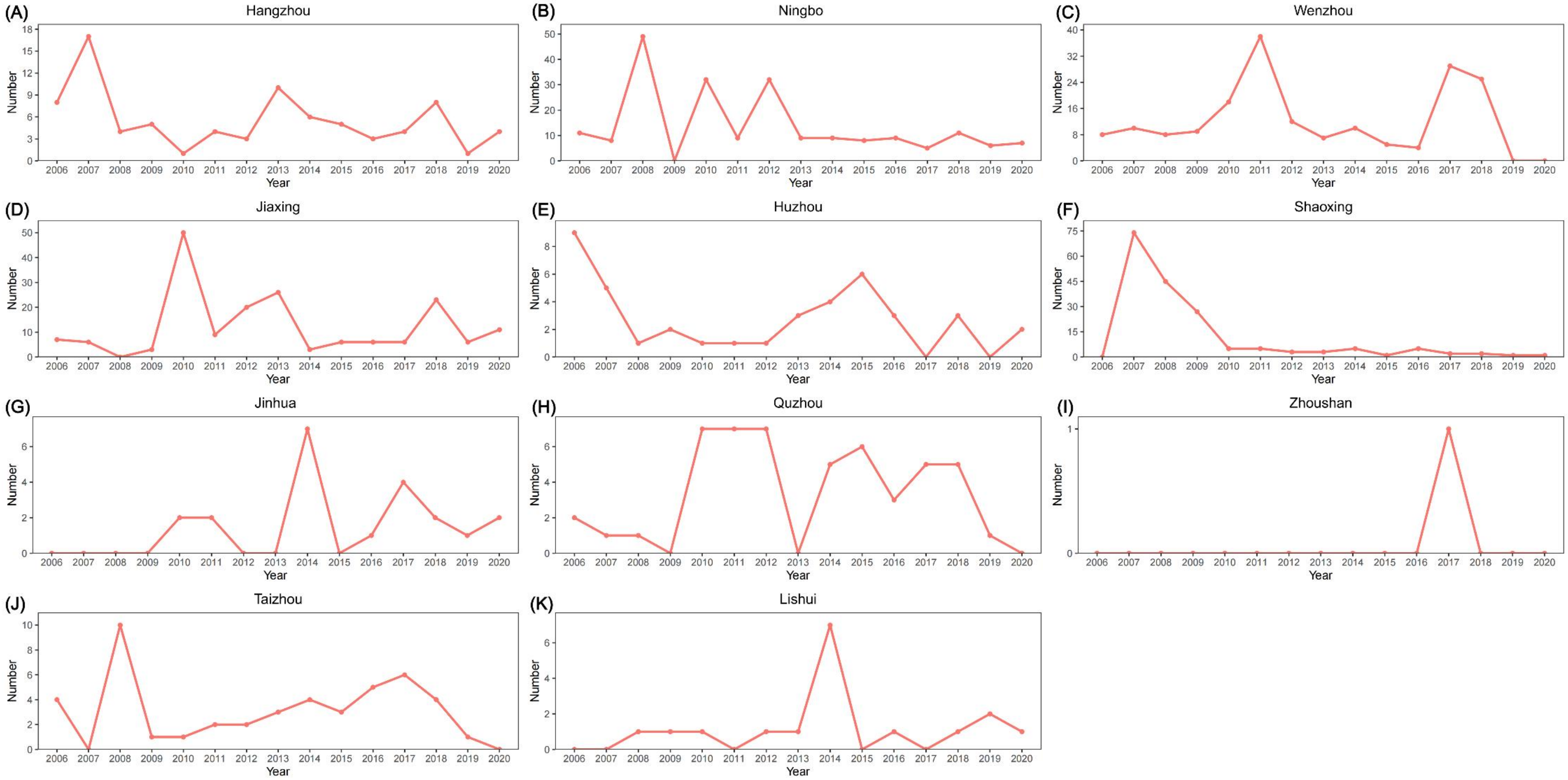

Figure S1. The changes in the number of occupational poisoning cases reported from 2006 to 2020 in Hangzhou (A), Ningbo (B), Wenzhou (C), Jiaxing (D), Huzhou (E), Shaoxing (F), Jinhua (G), Quzhou (H), Zhoushan (I), Taizhou (J), Lishui (K).

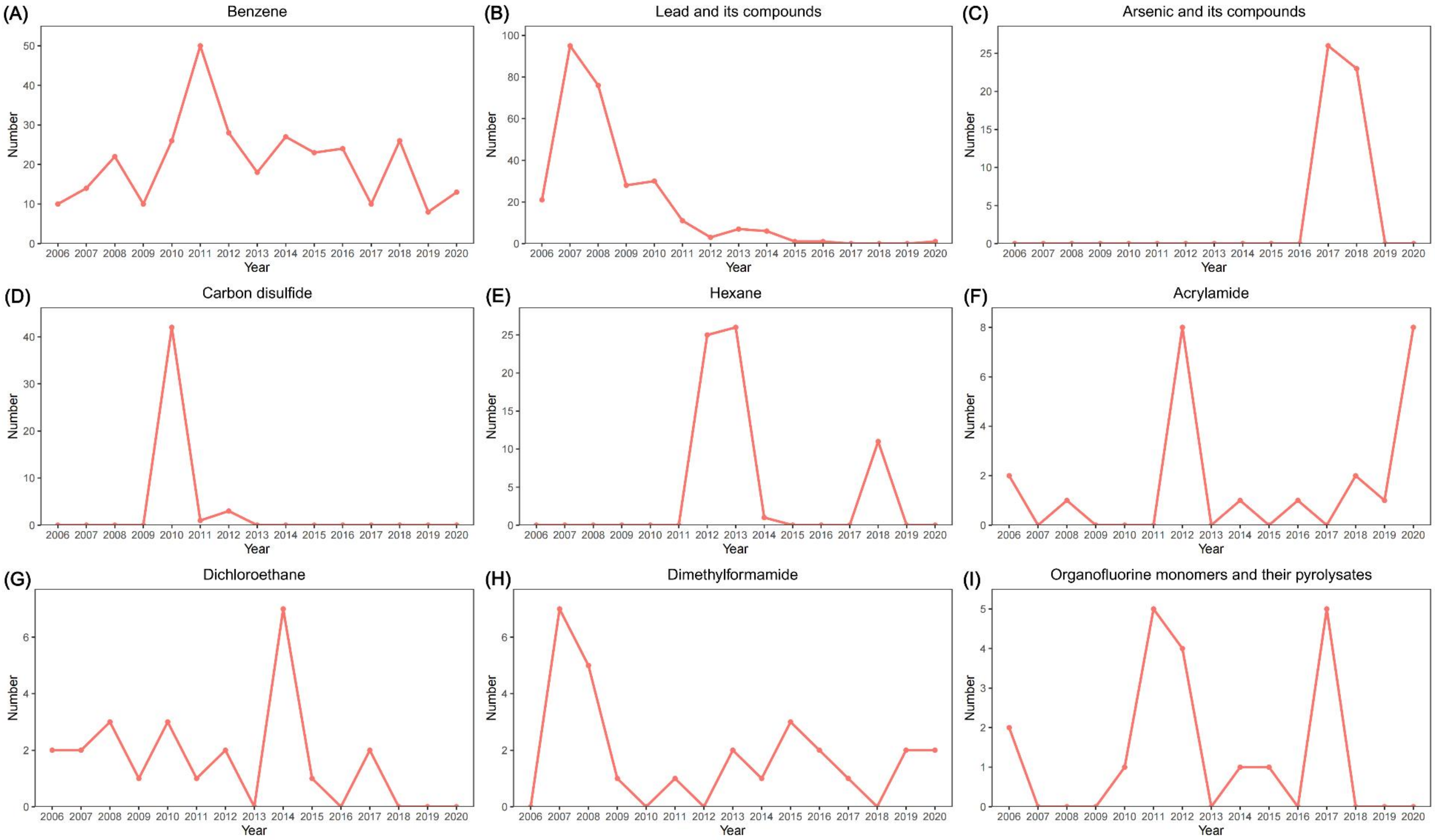

Figure S2. The changes in the number of reported cases of occupational poisoning by benzene (A), lead and its compounds (B), arsenic and its compounds (C), carbon disulfide (D), hexane (E), acrylamide (F), dichloroethane (G), dimethylformamide (H), organofluorine monomers and their pyrolysates (I) from 2006 to 2020.

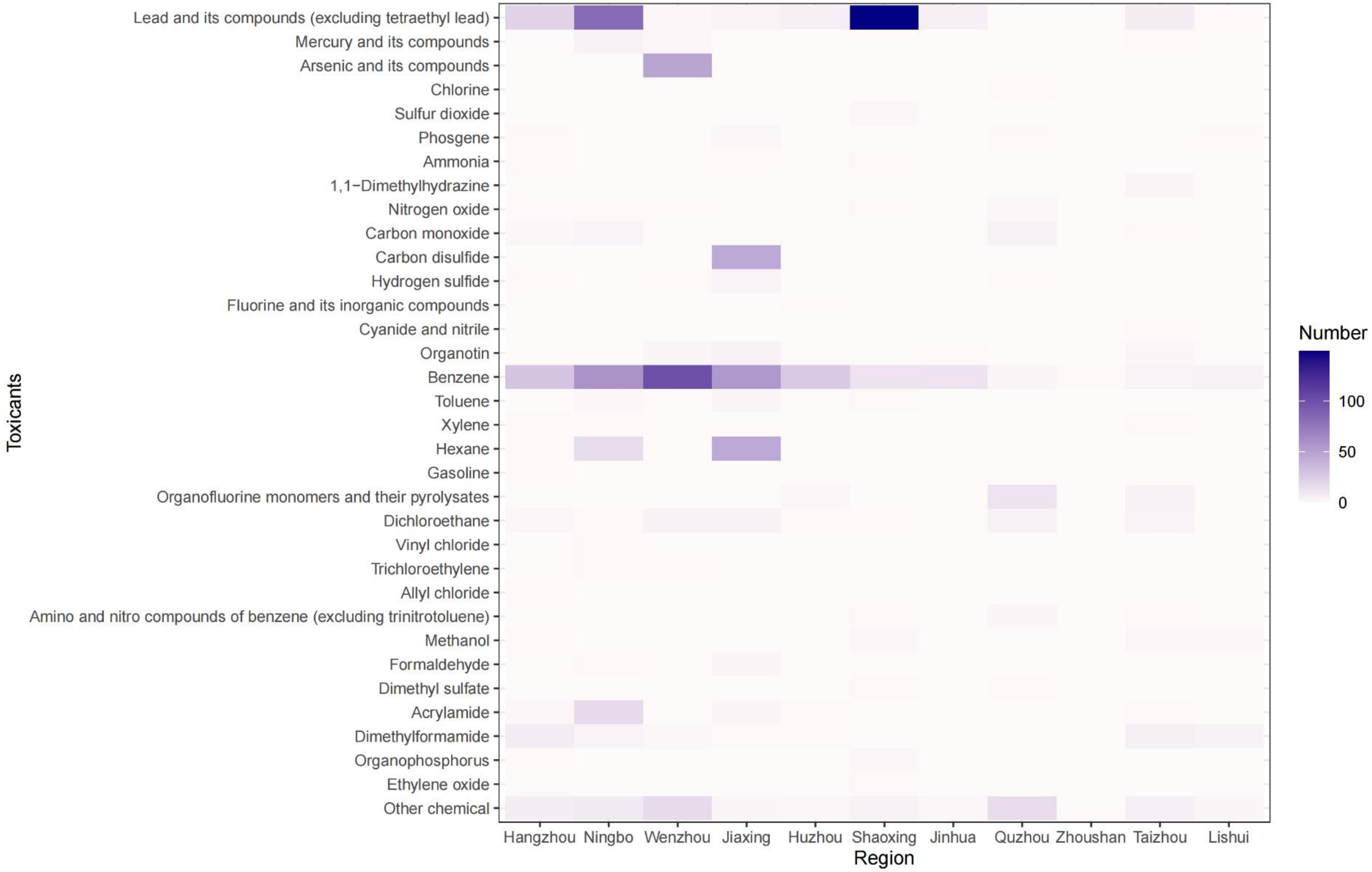

Figure S3. The regional distribution of toxicants causing occupational poisoning in Zhejiang Province from 2006 to 2020.

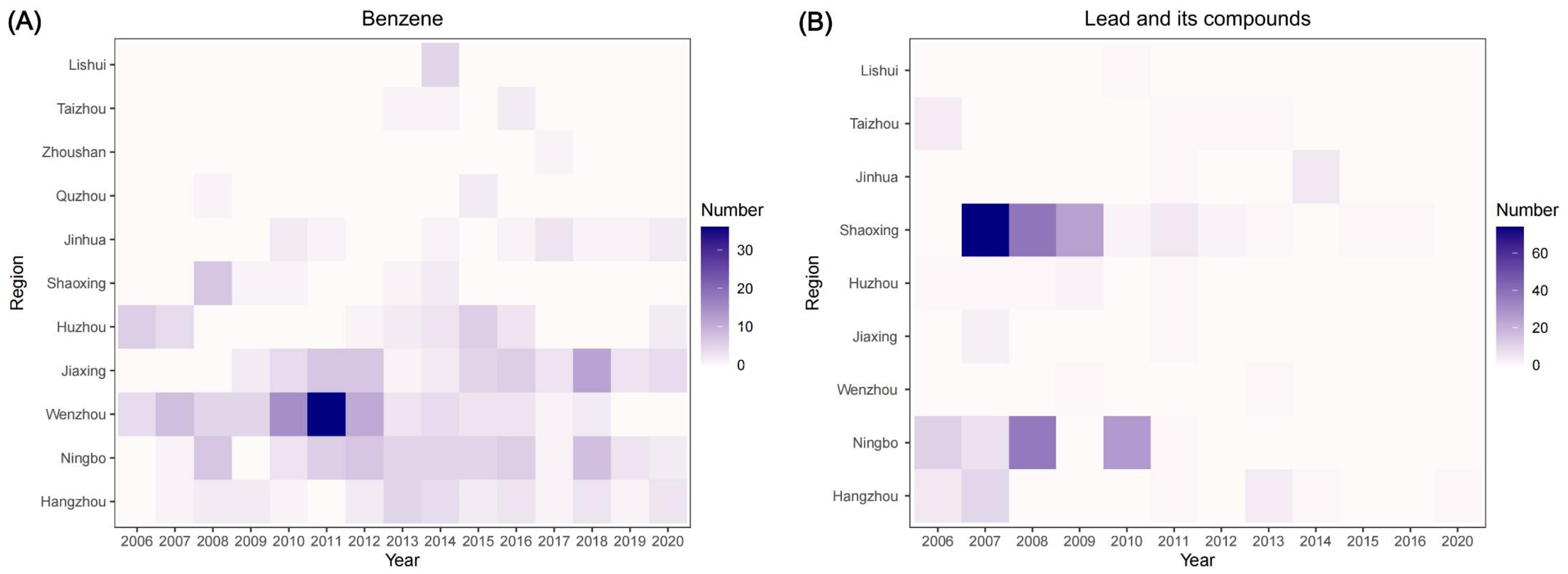

Figure S4. The regional distribution of occupational poisoning cases caused by benzene (A) and lead and its compounds (B) in Zhejiang Province from 2006 to 2020.

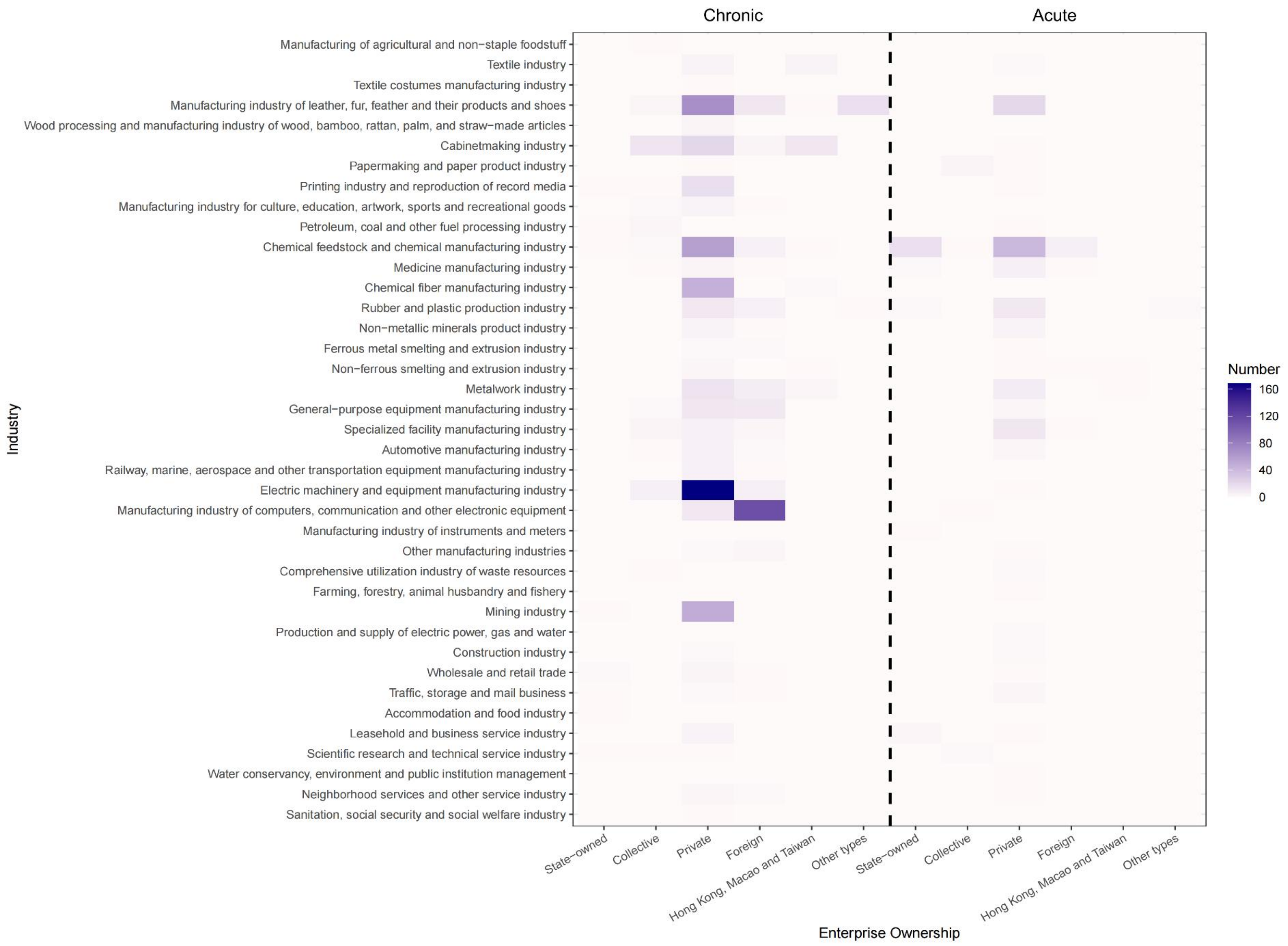

Figure S5. The enterprise ownership and industry distribution of occupational poisoning cases in Zhejiang Province from 2006 to 2020.

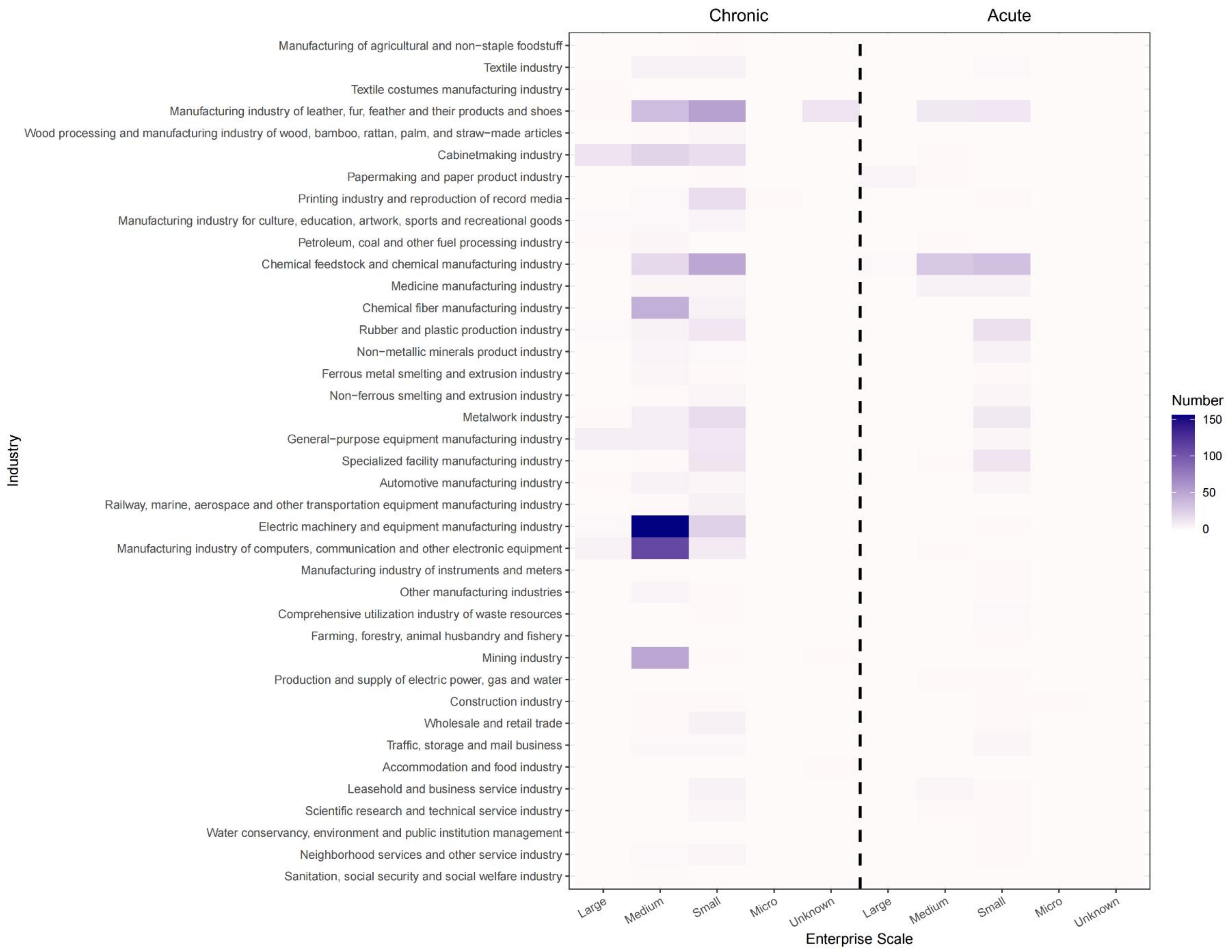

Figure S6. The enterprise scale and industry distribution of occupational poisoning cases in Zhejiang Province from 2006 to 2020.
